# Long-term outcomes after contaminated complex abdominal wall reconstruction

**DOI:** 10.1007/s10029-020-02124-7

**Published:** 2020-02-20

**Authors:** F. E. E. de Vries, J. D. Hodgkinson, J. J. M. Claessen, O. van Ruler, C. A. Leo, Y. Maeda, O. Lapid, M. C. Obdeijn, P. J. Tanis, W. A. Bemelman, J. Constantinides, G. B. Hanna, J. Warusavitarne, C. Vaizey, M. A. Boermeester

**Affiliations:** 1 Department of Surgery, Amsterdam University Medical Centres, University of Amsterdam, G4-133, PO Box 22660, 1100 DD Amsterdam, The Netherlands; 2 Department of Colorectal Surgery, St Marks Hospital, Academic Institute London, London, UK; 3 Department of Surgery and Cancer, Imperial College London, London, UK; 4 Department of Surgery, IJsselland Hospital, Capelle a/d IJssel, The Netherlands; 5 Department of Plastic and Reconstructive Surgery, Amsterdam University Medical Centres, University of Amsterdam, Amsterdam, The Netherlands

**Keywords:** Hernia repair, Contamination, Outcomes, Complex, Mesh

## Abstract

**Purpose:**

Complex abdominal wall repair (CAWR) in a contaminated operative field is a challenge. Available literature regarding long-term outcomes of CAWR comprises studies that often have small numbers and heterogeneous patient populations. This study aims to assess long-term outcomes of modified-ventral hernia working group (VHWG) grade 3 repairs. Because the relevance of hernia recurrence (HR) as the primary outcome for this patient group is contentious, the need for further hernia surgery (FHS) was also assessed in relation to long-term survival.

**Methods:**

A retrospective cohort study with a single prospective follow-up time-point nested in a consecutive series of patients undergoing CAWR in two European national intestinal failure centers.

**Results:**

In long-term analysis, 266 modified VHWG grade 3 procedures were included. The overall HR rate was 32.3%. The HR rates for non-crosslinked biologic meshes and synthetic meshes when fascial closure was achieved were 20.3% and 30.6%, respectively. The rates of FHS were 7.2% and 16.7%, and occurred only within the first 3 years. Bridged repairs showed poorer results (fascial closure 22.9% hernia recurrence vs bridged 57.1% recurrence). Overall survival was relatively good with 80% en 70% of the patients still alive after 5 and 10 years, respectively. In total 86.6% of the patients remained free of FHS.

**Conclusions:**

In this study of contaminated CAWR, non-crosslinked biologic mesh shows better results than synthetic mesh. Bridging repairs with no posterior and/or anterior fascial closure have a higher recurrence rate. The overall survival was good and the majority of patients remained free of additional hernia surgery.

## Introduction

Complex abdominal wall repair (CAWR) in a potentially contaminated operative field is a challenge. Sources of contamination such as the presence of a stoma, concomitant bowel surgery, enterocutaneous fistulas (ECF) or infected mesh increase the risk of wound complications [1, 2]. Additionally, patients may have coexisting intestinal failure, possibly worsening postoperative morbidity [3–6].

Available literature regarding outcomes of contaminated ventral hernia repair is sparse and often studies have small numbers and heterogeneous patient populations [7, 8]. Moreover, there is no standardization of outcome reporting. The choice of mesh remains controversial; synthetic and biologic crosslinked meshes are feared due to the risk of infection and fistulation while non-crosslinked biologic meshes are expensive, and long-term durability remains questionable [9, 10]. Because of these factors, a randomized controlled trial in this complex group of patients seems unachievable.

A recent systematic review [7] found 601 contaminated procedures described in 16 studies with an overall recurrence rate of 24.3% after an average follow-up period of 26.7 months. Hernia rates varied between studies, with huge heterogeneity in patients, mesh use and mesh positioning. Bridging repairs had a higher recurrence rate (40.0%) compared to repairs in which fascial closure was achieved (16.0%). Another systematic review, also including potentially contaminated procedures, found a weighted pooled recurrence rate of 9% for potentially contaminated procedures and 30% for contaminated procedures [8].

In contrast to clean or small hernias, this group of patients undergo months of complex wound and stoma care, and sometimes require home parenteral nutrition (PN). Therefore, the relevance of a recurrent hernia as an outcome measure, if small and asymptomatic, could be questioned.

In 2012, Kanters et al. [2] proposed the modified Ventral Hernia Working Group (VHWG) classification, replacing the original 2010 VHWG classification [1]. In this new classification, grade 3 and 4 hernias with (potential) contamination were combined to a modified VHWG grade 3. Patients with a previous wound infection, but no contamination at the time of surgery, were removed from the modified VHWG grade 3 group. A number of studies have assessed risk factors for hernia recurrence in this cohort. Factors such as active smoking, increased BMI, number of previous hernia repairs, number of previous abdominal surgeries and the use of bridging mesh were found to be significantly associated with hernia recurrence [11, 12].

This study aimed to assess long-term outcomes of modified VHWG grade 3 repairs in a combined cohort from a UK and a Dutch national referral center for intestinal failure. As the relevance of clinical hernia recurrence as the primary outcome for this complex and specific patient group is contentious, the need for further hernia surgery was also assessed in relation to overall survival.

## Material and methods

### Study design and data collection

This was a retrospective cohort study with a single prospective follow-up time-point nested in a consecutive series of patients undergoing CAWR in two European centers with nationwide referral for intestinal failure. All patients with modified VHWG grade 3 defects (clean-contaminated, contaminated or dirty) [2], operated in either the Amsterdam University Medical Centres (AUMC), location AMC, Amsterdam, the Netherlands or St Marks Hospital (SMH), London, United Kingdom, between 2004 and 2015 were identified. The VHWG grade 3 group involves patients with a stoma, concomitant bowel surgery, enterocutaneous fistulas (ECF) or infected mesh. The study protocol was approved by the medical ethical committees of both hospitals. All patients signed informed consent prior to participation and data were stored in a secured database.

Baseline characteristics on patient demographics, surgical procedures, and postoperative follow-up were collected by retrospective case note review. All patients still alive were invited to participate in a single out-patient clinic visit. If patients were not able to visit, they were requested to answer a questionnaire regarding long-term follow-up by telephone. If patients did not respond within 4 weeks, a second invitation to participate was sent. For patients who were not reachable or unable to attend, their last confirmed abdominal examination performed at the out-patient clinic was used as their last abdominal follow-up. The following definitions were used: overall survival (last moment the patient was known to be alive), hernia free survival (either the last moment a physician performed an abdominal examination without finding a recurrent hernia or the last known abdominal imaging without a hernia) and hernia-related-surgery free survival (last day before the patient underwent new hernia repair). If a patient had a midline laparotomy for another indication then hernia repair, patients were censored on the day of surgery.

### Outcome and definitions

The operative reconstruction was categorized by primary suture repair, synthetic mesh or non-crosslinked biologic mesh. For biologic mesh, these were further classified into biologic only, biologic in combination with synthetic absorbable mesh (SAM), and biologic in combination with synthetic non-absorbable mesh (SNAM). For synthetic mesh, these were further classified as SAM, SNAM and a combination of SAM and SNAM. Primary outcome was overall hernia recurrence. Hernia recurrence was further classified as clinically symptomatic (either needing surgery or reported as symptomatic by the patient) or asymptomatic. Additional surgery for hernia recurrence (interventional outcome) was classified as surgery for a recurrent clean hernia or for a recurrent (potentially) contaminated hernia. The results were stratified by fascial closure achieved repairs and bridged repairs. Intestinal failure (IF) is defined as the inability of the intestinal tract to maintain protein/energy, fluid, electrolyte or micronutrient balance resulting in the need for intravenous (iv) fluid supplementation and/or total parenteral nutrition (TPN) [13]. The ability to wean off PN within 2 years after reconstructive surgery was also recorded, as recommended by the European Society of Coloproctology (ESCP) intestinal failure guidelines [13]. Patients visiting the out-patient clinic were asked to perform the Trunk-raising test [14] to test their abdominal muscle strength (straight sit-up on a 5-point scale, “Appendix”).

### Surgical technique

Both centers have a long-standing history of treating patients with contaminated abdominal wall defects and intestinal failure, and operative strategies were comparable between the centers and consistent with published guidelines and consensus [13].

Peri-operative infection prevention measures were used according to local standards and included appropriate antibiotic prophylaxis administration before incision, hand-hygiene, and surgical site preparation with an alcohol and chlorhexidine based anti-septic agent. At the AUMC, closed incision negative pressure wound therapy was introduced in January 2014. Both centers had a preference for planned admission to a high dependency unit for at least 24 h postoperatively.

Operative goals were driven by best practice evidence at the time of surgery. Briefly, according to consensus on the management of patients with intestinal failure; enterocutaneous fistulas were resected and, usually, a primary hand sewn anastomosis was performed; number of anastomoses was minimized; a double layer anastomosis was preferred; and up-stream diverting stomas were used whenever necessary. The primary aim of repair of the abdominal wall defect was tension-free fascial closure with reinforcing mesh where possible. Component separation techniques (CST) were used if required and possible. Plastic and reconstructive surgeons were consulted and performed reconstruction in cases of large full thickness skin defects and those with significant loss of domain.

Choice of mesh type was tailored to the individual patient but based on some basic principles. In defects with minimal contamination (for example stoma present) and a defect that could be closed after CST, a SNAM could be used. If there was gross contamination, biologic, SAM or no mesh was preferred. Up until 2011, SAM (polyglactin) was preferred in both centers, after this, a non-crosslinked biological mesh became the primary choice, preferably in an intra-abdominal (IPOM) or retro-rectus (sublay) position. In some cases, with particularly large defects, a double or even triple layer mesh technique was employed based on surgeon choice.

Fascial closure was typically performed using a looped monofilament polydioxanone (PDS) suture. If fascial closure was not achievable, the defect was bridged using an intra-abdominal or retro-muscular mesh or both. Passive intra-abdominal drains were used where deemed appropriate but not routinely, and active subcutaneous drains were used routinely. Drains were removed when output was below 30 ml/24 h. No skin grafts were used to achieve primary skin closure. AUMC preferred interrupted polyester Mersilene sutures (Ethicon), whereas SMH tended to use surgical clips or interrupted polypropylene prolene sutures (Ethicon). If a plastic surgeon was involved layered closure was performed with absorbable sutures subcutaneous.

It was noted that post-operative wound infections/collections were managed differently between the two centers. AUMC had a preference for performing radiological percutaneous drainage of wound collections, whereas SMH routinely opened wounds at the bedside to drain collections.

### Statistical analysis

Baseline characteristics are presented as numbers and percentages. Normally distributed data is presented as a mean with standard deviation (SD), while non-normally distributed data is presented as a median with interquartile range (IQR) or total range. Overall survival, hernia free survival, and hernia-related-surgery free survival were assessed using Kaplan Meier statistics and compared using a Log-rank test. Binary logistic regression was performed to identify risk factors associated with long-term complications. A *p* value of < 0.05 was considered significant.

## Results

A total of 272 VHWG grade 3 contaminated abdominal wall reconstructions performed between 2004 and 2015 in 254 patients were included in the study. Mean age was 58.0 (SD 13.6) and 58.8% were male. Patient and operative characteristics are presented in Table 1. Six of the initial 254 patients (2.4%) died in-hospital and were therefore excluded from long-term assessment of hernia recurrence and re-repair, leaving 266 procedures in 248 patients.

**Table 1:** Patient and operative characteristics of 272 modified VHWG grade 3 procedures in 254 patients

	272 procedures in 254 patients Percentage of cohort (*N*)
Patient characteristics	
Age, mean (SD)	58.0 (SD 13.6)
Sex male	58.8% (160)
BMI	Median 26.0 (IQR 22.6–29.6)
ASA classification, mean (SD)	Mean 2.43 (SD 0.5)
2	59.6% (162)
3	37.9% (103)
4	2.6% (7)
Active smoker	22.8 (62)
Diabetes	18.4 (50)
Immunosuppression	7.7 (21)
Cardiac comorbidity,	23.2 (63)
Pulmonary comorbidity	20.2 (55)
COPD	10.7 (29)
Hypertension	30.5 (83)
IBD	14.3 (39)
Intestinal failure	47.1 (129)
Previous abdominal malignancy	18.4 (50)
History of open abdomen	47.1 (128)
Presence of intestinal fistula	58.1 (159)
Operative characteristics	
Number of previous abdominal surgeries, median	4 (IQR 2–5) (range 1–25)
Undergone previous hernia repairs	43.8 (119)
Time since last surgery	Median 349 days (IQR 240–636)
CDC wound classification	
2	30.1 (82)
3	45.6 (124)
4	24.3 (66)
Anastomosis constructed	73.5 (200)
Mesh removal	21.7 (59)
Component separation technique performed	
Yes	67.3 (183)
No	31.6 (86)
Unknown	1.1 (3)
Mesh used	66.9 (182)
Fascial closure	
Yes, fascial closure without mesh	32.4% (88)
Yes, reinforcement with mesh	40.1% (109)
No, bridging with mesh	24.6% (67)
Unclear	2.9% (8)

*BMI* body mass index, *ASA *American Society of Anesthesiologists, *COPD *chronic obstructive pulmonary disease, *IBD *inflammatory bowel disease, *CDC *center for disease control and prevention

### Survival

**Fig. 1:**
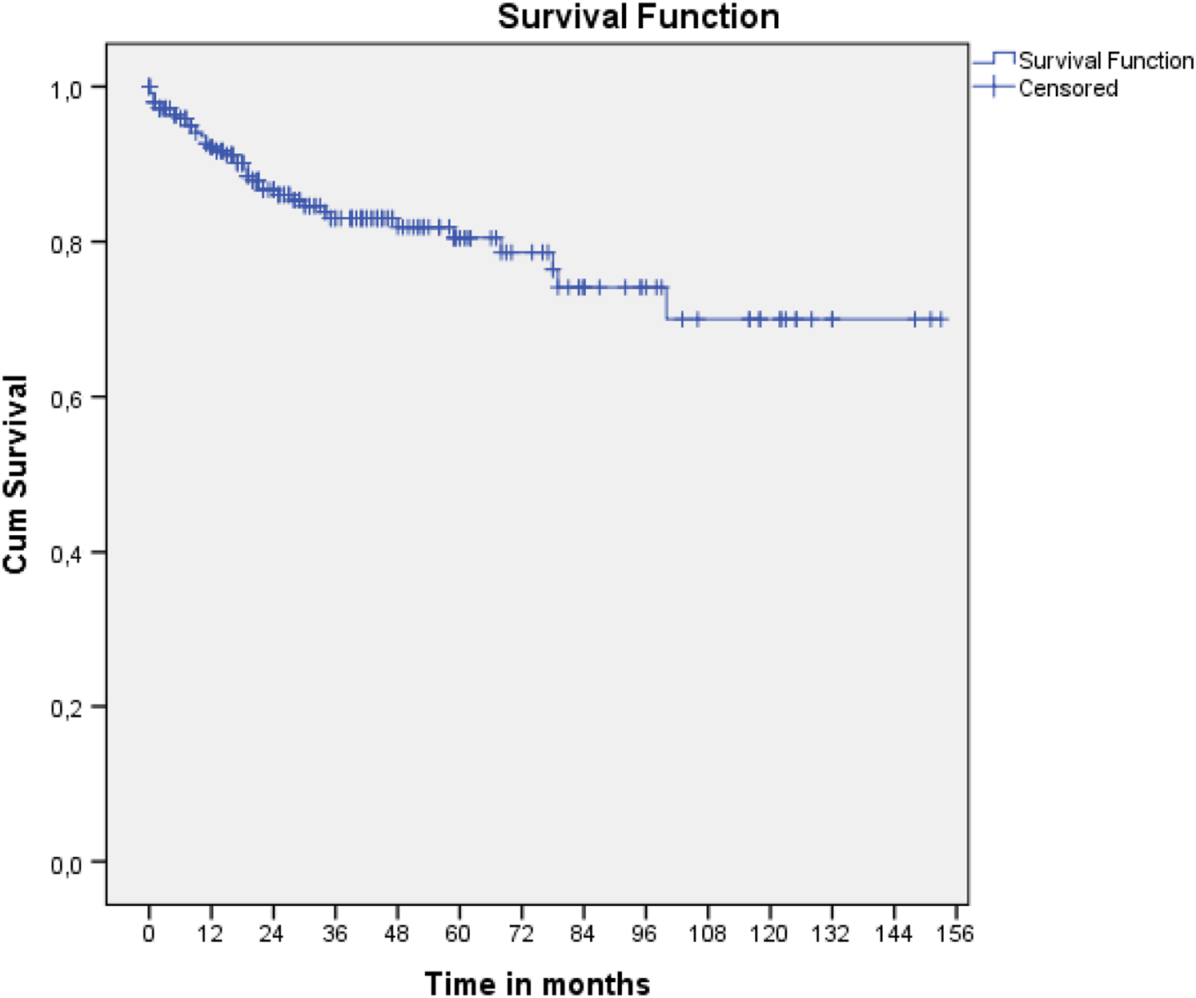
A Kaplan–Meier depicting overall survival of the cohort at long-term follow-up. Survival is 80% at 5 years and 70% at 10 years

One-year mortality was 8.7% (22 patients including the in-hospital deaths (6)). After a mean follow-up of 36 months (range 0–153 months), another 21 patients died. Therefore, 211 of 254 patients (83.1%), who underwent a total of 229 procedures, were still alive at the time the study was performed. Actuarial 5-year and 10-year overall survival probabilities were 80% and 70%, respectively (Fig. 1).

### Long-term follow-up

211 patients were invited to participate in a long-term follow-up out-patient clinic. We had a response rate of 71.1% (150 patients), and only 9 of those patients were unwilling to participate. Between October 2016 and March 2017, 99 patients were seen at the out-patient clinic, and 42 patients answered a questionnaire by telephone contact. For the remaining patients, we used the last available information from the medical records.

### Use of mesh and mesh mediated risk factors

The choice of reconstruction technique was made by the attending surgeon based on the principles as described in our surgical technique section. Table 2 demonstrates the operative challenges and the corresponding choice of mesh. As shown in Table 2, SNAM were mostly used in procedures with low risk of contamination, while SAM (until 2011) and non-crosslinked biologic mesh (available from 2011 in both hospitals) were mostly used in procedures with high risk of contamination.

**Table 2:** A comparison of operative challenges and choice of mesh (*n* = 272) in all cases

	ECF with infected mesh	ECF without infected mesh	Infected mesh	Violation of the GI-tract^a^	Stoma present	Other	Total (%)
No mesh	13	38	5	34	0	0	90 (33.1)
Biologic^b^	22	44	9	21	1	1	98 (36.0)
Biologic + SAM^c^	2	3	0	1	0	0	6 (2.2)
Biologic + SNAM^d^	0	1	1	2	1	1	6 (2.2)
SNAM^e^	1	2	1	5	10	3	22 (8.1)
SAM^f^	2	25	2	5	0	1	35 (12.9)
SNAM + SAM^g^	1	4	0	7	1	2	15 (5.5)
Total (%)	41 (15.0)	117 (43.0)	18 (6.6)	75 (27.6)	13 (4.8)	8 (2.9)	272 (100)

*ECF *enterocutaneous fistula, *GI* gastrointestinal, *SAM* synthetic absorbable mesh, *SNAM* synthetic non-absorbable mesh

^a^for example bowel resection, enterostomy creation or anastomosis

^b^Mesh used: Strattice

^c^Mesh used: Strattice, vicryl

^d^Mesh used: Strattice, Vypro

^e^Mesh used: Vypro, Ultrpro, Physiomesh, Surgisis, Proceed, Dualmesh, Prolene

^f^Mesh used: Vicryl

^g^Mesh used: Vypro, Vicryl

Mesh use, mesh type, and mesh position were further evaluated (Table 3). SAM or SNAM use was associated with an increased risk of recurrence and further surgery compared to biologic mesh. Bridging mesh was found to have a higher risk of symptomatic hernia recurrence compared to no mesh or the use of mesh as reinforcement [OR 4.22 (95% CI 1.53–11.60), *p* ≤ 0.01)]. The use of an additional mesh in high-risk patients was associated with an increased risk of hernia recurrence [OR 2.24 (95% CI 1.06–4.70), *p* = 0.03]. Surgery for hernia recurrence was more likely when a mesh was used in an onlay or bridging position (8.0% after fascial vs 25.7% after bridged closure).

**Table 3:** Mesh associated risk factors for hernia recurrence and further hernia surgery

	Hernia recurrence		Further hernia surgery	
	OR (95%CI)	*p* value	OR (95%CI)	*p* value
Mesh type				
Biologic	1.27 (0.66–2.46)	0.48	2.40 (0.82–7.02)	0.11
SNAM	2.30 (0.95–5.57)	0.07	6.11 (1.82–20.53)	< 0.01
SAM	5.28 (2.40–11.61)	< 0.01	4.45 (1.39–14.24)	0.01
Mesh position				
Sublay IA	1.90 (0.99–3.65)	0.06	2.98 (1.03–8.65)	0.05
Retrorectus	1.97 (0.72–5.37)	0.18	2.65 (0.58–12.07)	0.21
Onlay	1.79 (0.79–4.04)	0.16	4.07 (1.24–13.36)	0.02
Bridging IA sublay	4.22 (1.53–11.60)	< 0.01	5.60 (1.44–21.73)	0.01
Additional mesh	2.24 (1.06–4.70)	0.03	1.55 (0.62–3.83)	0.35

*IA* intra-abdominal, *SAM* synthetic absorbable mesh, *SNAM* synthetic non-absorbable mesh

### Enterocutaneous fistula

Twenty patients developed an ECF postoperatively (7.5%). These were 18 recurrent and 2 new fistulas. Twelve patients had additional surgery for their (recurrent) fistula, and seven developed a long-term stable low-output fistula. One patient had spontaneous closure of the fistula after long-term conservative management.

### Hernia recurrence and additional surgery for hernia recurrence

**Fig. 2:**
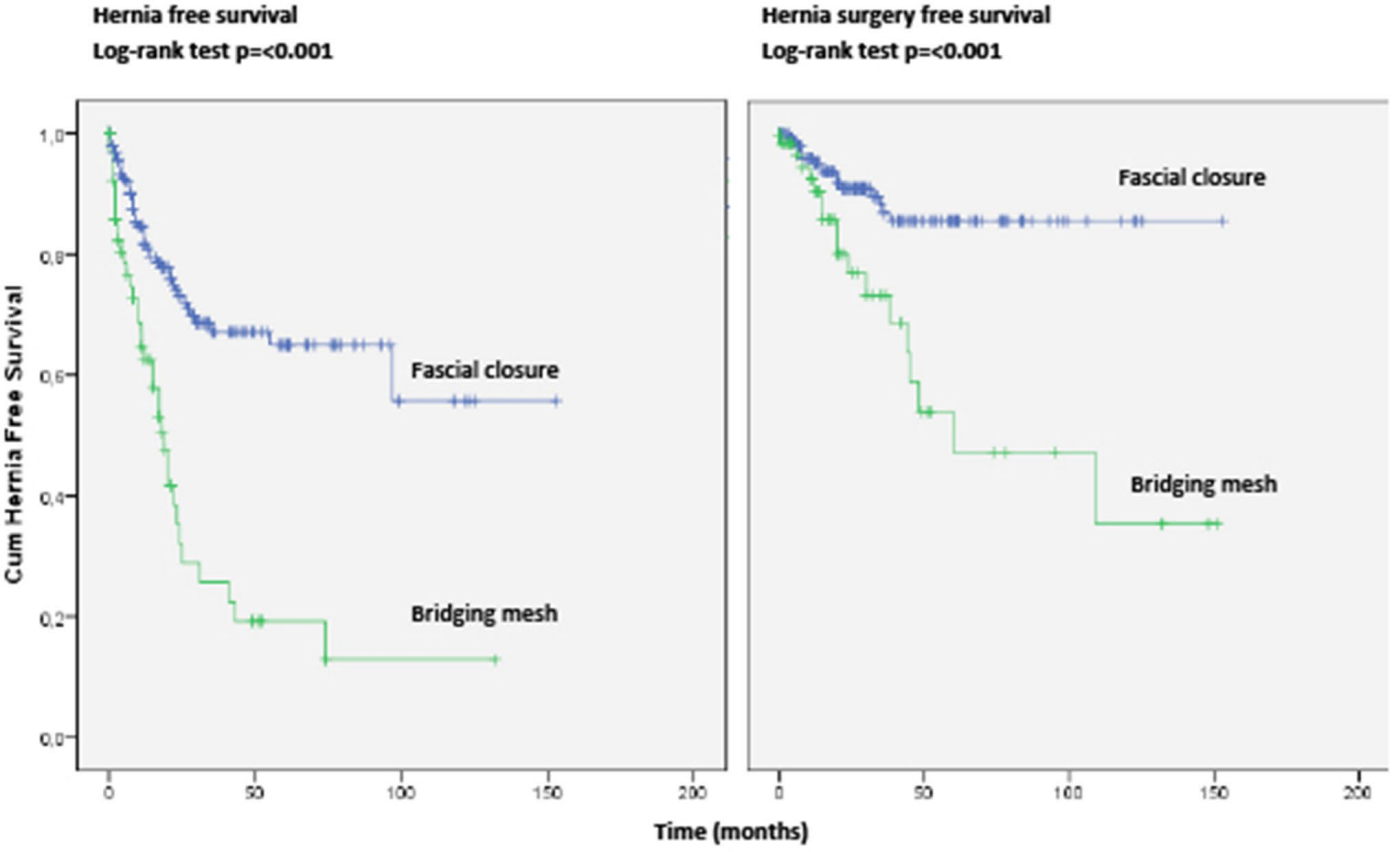
A Kaplan–Meier depicting hernia free and hernia surgery free survival. Log rank test demonstrates a significant difference between fascial closure achieved repairs and bridged repairs for both outcomes

A total of 86 of the 266 (32.3%) procedures resulted in a recurrent hernia during follow-up. Thirty-six of these, 86 recurrences (13.5% of the total procedures and 41.9% of the recurrences) underwent additional hernia surgery. Table 4 shows hernia recurrence rates and surgery for hernia recurrence rates for both repairs with fascial closure and bridging repairs, and for different types of mesh. The hernia recurrence rates were 22.9% and 57.1% for fascial closure and bridging repair, while the rate of further hernia surgery were 8.0% and 25.7%, respectively. In selected cases with primary suture repair, 21.7% had a hernia recurrence and 4.8% underwent further hernia surgery. Biologic mesh showed fewer HR and further hernia surgery, both in bridged and facial closure repairs. Bridged repairs demonstrated poorer results, with a 45.7% recurrence rate for biologic mesh and 75.0% for synthetic mesh. Of these, 25.7% of the biologic mesh group underwent further hernia surgery compared to 28.1% in the synthetic mesh group. Figure 2 shows Kaplan Meier curves of hernia-free survival and hernia-related-surgery free survival, respectively. Log-rank tests showed a significant difference for both hernia recurrence and further hernia-related-surgery (*p* < 0.001) when comparing fascial closure with a bridged repair. Kaplan Meier regression showed that in patients where fascial closure was achieved, 86.6% were free from further surgery at 3 and 10 years.

**Table 4:** Long-term follow-up data demonstrating overall hernia recurrence and further hernia related surgery and divided by repair technique (no mesh, biologic mesh, synthetic mesh) and by ability to achieve fascial closure

	Hernia recurrence			Additional surgery related to hernia recurrence		
	No bridging	Bridging	Unknown	No bridging	Bridging	Unknown
No mesh	18/83 = 21.7%	0/3	2/3	4/83 = 4.8%	0/3	2/3
Biologic total	14/69 = 20.3%	16/35 = 45.7%	0/2	5/69 = 7.2%	9/35 = 25.7%	0/2
Biologic only^a^	13/63 = 20.6%	10/29 = 34.5%	0/2	5/63 = 7.9%	6/29 = 20.7%	0/2
Biologic + SAM^b^	1/4	2/2	–	0/4	0/2	–
Biologic + SNAM^c^	0/2	4/4	–	0/2	3/4	–
Synthetic total	11/36 = 30.6%	24/32 = 75%	1/3	6/36 = 16.7%	9/32 = 28.1%	1/3
SNAM^d^	5/16 = 31.3%	4/5	0/1	3/16 = 18.8%	2/5	0/1
SAM^e^	5/17 = 29.4%	15/17 = 88.2%	–	3/17 = 17.6%	4/17 = 23.5%	–
SNAM + SAM^f^	1/3	5/10 = 50.0%	1/2	0/3	3/10 = 30.0%	1/2
Total	43/188 = 22.9%	40/70 = 57.1%	3/8	15/188 = 8.0%	18/70 = 25.7%	3/8

*SAM* synthetic absorbable mesh, *SNAM* synthetic non-absorbable mesh

^a^Mesh used: Strattice

^b^Mesh used: Strattice, vicryl

^c^Mesh used: Strattice, Vypro

^d^Mesh used: Vypro, Ultrpro, Physiomesh, Surgisis, Proceed, Dualmesh, Prolene

^e^Mesh used: Vicryl

^f^Mesh used: Vypro, Vicryl

### Discontinuation of parenteral nutrition

At the time of reconstructive surgery, 125 patients (47.1%) of the present cohort depended on PN. We found that 104 patients (80.6%) successfully weaned of PN after a median of 54 days (range 1–562 days). Three patients died before they were able to wean PN. Therefore, 16 patients were still dependent on PN 2 years after surgery. Of those, six patients had an absolute short-bowel, four patients had a recurrent fistula, two patients had an atonic bowel segment and were therefore unable to discontinue PN, two patients remained on intravenous fluid supplementation only, one developed metastatic lung cancer, and one patient was unfit for reversal of a high stoma.

### Trunk-raising test

All patients visiting the out-patient clinic were asked to perform the Trunk-Raising test [14]. Seventy patients performed the test and their median score was 5 (IQR 3–5) which is the maximum score on the test.

## Discussion

Present study shows that, although overall hernia recurrence rates after contaminated CAWR are high, the majority of patients do not need to undergo further surgery, with an 80% 5-year overall survival rate. The overall hernia recurrence rate of 32.3% is comparable with literature [15], and results were worse in bridged repairs compared to repairs in which fascial closure could be achieved. Synthetic mesh use was associated with higher recurrence rates, although the operative challenge in these cases seems to have been less as shown by the level of contamination. When fascial closure was achieved, further hernia surgery was performed in the first 3 years and 86.6% of the patients never underwent further hernia surgery.

Previous studies [16–20] showed worse results for bridged repairs compared to fascial closure achieved repairs. Our data are in line with literature and also reveals poor results for both hernia recurrence and for additional surgery when a bridged repair was used, with a quarter of the patients with recurrence undergoing further hernia surgery during follow-up (Fig. 2). Therefore, every effort should be made to achieve fascial closure.

Although the results of primary suture repair in this cohort are comparable to the use of biologic mesh, this is probably due to selection of less complex defects in which tension-free fascial closure could be achieved and a mesh not considered of additional value by the operating surgeon. This is supported by risk factor analysis, demonstrating that mesh was associated with recurrence, implying that mesh is more likely to be used in high risk cases. A previous study [17] has shown that primary suture repair or CST without mesh reinforcement have high recurrence rates. Therefore a reasonable indication for biologic mesh could be to allow fascial closure under tension and better distribution of forces in the midline, thereby achieving fascial closure even if it was deemed impossible prior to the application of biologic mesh.

Evaluation of mesh type and position revealed an apparent advantage of biologic mesh in long-term outcomes in comparison with synthetic meshes. Controversy exists regarding the choice of mesh in a contaminated setting. A recent systematic review and meta-analysis [8] including 32 studies showed comparable results for synthetic and biologic meshes in terms of hernia recurrence for potentially contaminated repairs, but higher recurrence rates were seen in contaminated repairs using biologic mesh. A more recent paper questions the use of biologic mesh altogether if compared to synthetic mesh, but highlighting the very low level of evidence in the field of CAWR [21]. Our data clearly show that there is an inevitable bias in comparing synthetic and biologic mesh. As shown in Table 2, most non-absorbable meshes were used in procedures with a relatively small risk of contamination (such as a stoma present), whereas most non-crosslinked biologic meshes were used for ECF or infected mesh surgery. Despite this selection bias, our data show better results for biologic mesh, both in hernia recurrence rates and re-repair rates. In other words, use of non-crosslinked biologic meshes could be beneficial in complex situations. A possible explanation for the higher incisional hernia recurrence rates for synthetic mesh might be that more synthetic non-absorbable mesh needed to be removed due to infectious complications.

Biologic mesh is currently recommended in guidelines [1, 13] for contaminated repairs, but costs are high and, therefore, the use of biologic mesh has sometimes been questioned. Long-term gains outweigh the initial high cost, considering that the overall survival in this group is good and the majority of patients do not undergo any further surgery and are subsequently weaned of PN. In this series, only non-crosslinked biologic mesh were used as several studies show poor results with cross-linked biologic mesh with high numbers of surgical site occurrence, mesh removal and fistulation [9, 10, 21–23]. However, appropriate patient selection for the use of a non-crosslinked biologic mesh is of utmost importance, and studies to assess cost-effectiveness are needed.

Present study has several limitations. Most of the data was retrospectively collected and not all patients were available for long-term follow-up. However, as much information as possible was gathered by clinical examination, questionnaires and telephone interviews. Radiologic confirmation of a recurrent hernia was not achieved for all cases as we did not routinely perform CT follow-up; we only used available CTs in this study. A recent study demonstrates that incisional hernia cannot be diagnosed by patient-reported diagnostic questionnaire but might be used to rule out incisional hernia [24]. In addition, in this study, we faced the problem that the population of patients undergoing contaminated CAWR is heterogeneous and sometimes combination of meshes were used for the repair. Exclusion of the patients with combinations of meshes would not reflect daily practice, while choosing to make more groups results in very fragmented and small groups making it difficult to draw any conclusions. Therefore, we chose to include these patients and show the type of meshes used in the tables (Tables 2, 4). Other limitations included the fact that the cause of death after hospital discharge in most patients was unknown. This is because many patients were referred from external centers and, therefore, this data was impossible to obtain without informed consent. Although unlikely, it remains unknown whether long-term mortality is related to the CAWR. In assessing hernia-related-surgery free survival there is a bias, as patients deemed inoperable are not taken into account.

Despite these limitations, this is a large cohort of modified VHWG grade 3 procedures managed by the same treatment principles from two dedicated complex AWR and intestinal failure centers. The data suggest that bridged repairs should be avoided if possible and that non-crosslinked biologic mesh shows better results than synthetic mesh in contaminated CAWR.

As 80% of patients are still alive at 5-year follow-up and 86.6% of the patients remained free of additional surgery, the initial higher costs of a non-crosslinked biologic mesh seem trivial.

## Appendix: Trunk-raising test

The patient lies in a supine position with the hips flexed at 45° and the knees at 90°. The examiner asks the patient to perform a curl-up by raising both the head and the shoulder blades from the table and holding this position for a minimum of 20 s. The score for this test is determined by the arm position the patient uses to elevate the scapulae off the table while the time the patient is able to hold this position is measured.

For a maximum score, the hands are to be clasped behind the head, and the position needs to be maintained for 20 s. If this is not feasible for the patient, the second option is to cross the arms in front of the body. The third option is to have both arms reaching out in front of the body. The potential scores are as follows:

- Hands behind neck, scapulae clearing the table, 20-s hold (normal) (5 points).
- Arms crossed over chest, scapulae clearing the table, 20-s hold (good) (4 points).
- Arms straight, scapulae clearing the table, 10-s hold (fair) (3 points).
- Arms extended toward knees, top of scapulae lifting from the table (poor) (2 points).
- Inability to raise more than head off the table (trace) (1 point).
